# Copy-number changes in evolution: rates, fitness effects and adaptive significance

**DOI:** 10.3389/fgene.2013.00273

**Published:** 2013-12-10

**Authors:** Vaishali Katju, Ulfar Bergthorsson

**Affiliations:** Department of Biology, University of New MexicoAlbuquerque, NM, USA

**Keywords:** copy-number variants, deletion, duplication, fitness effect, spontaneous rate

## Abstract

Gene copy-number differences due to gene duplications and deletions are rampant in natural populations and play a crucial role in the evolution of genome complexity. Per-locus analyses of gene duplication rates in the pre-genomic era revealed that gene duplication rates are much higher than the per nucleotide substitution rate. Analyses of gene duplication and deletion rates in mutation accumulation lines of model organisms have revealed that these high rates of copy-number mutations occur at a genome-wide scale. Furthermore, comparisons of the spontaneous duplication and deletion rates to copy-number polymorphism data and bioinformatic-based estimates of duplication rates from sequenced genomes suggest that the vast majority of gene duplications are detrimental and removed by natural selection. The rate at which new gene copies appear in populations greatly influences their evolutionary dynamics and standing gene copy-number variation in populations. The opportunity for mutations that result in the maintenance of duplicate copies, either through neofunctionalization or subfunctionalization, also depends on the equilibrium frequency of additional gene copies in the population, and hence on the spontaneous gene duplication (and loss) rate. The duplication rate may therefore have profound effects on the role of adaptation in the evolution of duplicated genes as well as important consequences for the evolutionary potential of organisms. We further discuss the broad ramifications of this standing gene copy-number variation on fitness and adaptive potential from a population-genetic and genome-wide perspective.

## INTRODUCTION

The publication of Ohno’s “*Evolution by Gene Duplication”* is fittingly viewed as a milestone in the study of gene duplications (Ohno, 1970). In addition to collating evidence for duplications in evolution, it also presented several hypotheses that have since been undergoing robust testing and analyses. For example, Ohno perceived that segmental duplications would be associated with problems with gene dosage balance and genetic instability, and therefore he also placed a great significance on whole-genome duplications. Additionally, he viewed the duplicate copy of a gene as an initially passive element in the evolution of new genes. A duplicated gene was seen as superfluous and therefore not under selection after duplication, that is, not until subsequent mutations conferred novel beneficial functions. Therefore, Ohno predicted that in the majority of instances, a gene duplicate would be lost or degenerate into a pseudogene.

The first characterized segmental gene duplication was the *bar* mutation in *Drosophila melanogaster* (Sturtevant, 1925). Soon after the discovery of the *bar* mutation, Bridges (1935, 1936) suggested that the duplication of genes provided a mechanism for increasing chromosome length and providing material for subsequent functional changes. This potential borne by gene duplication for evolutionary change was further emphasized by early geneticists and evolutionary biologists like Haldane, Müller, and Huxley (Haldane, 1933; Müller, 1935, 1936; Huxley, 1942). The *bar *mutation also serves as an illustration of several general features that should be emphasized about duplications. First, although it is “simply” a duplication of previously existing material that is expected to increase “redundancy” in the genome, the duplication has a striking phenotype. Gene duplication theory often treats duplications as having no immediate consequences after conception under the general assumption that gene duplicates must endure a passive existence in the genome until subsequent mutational events shape their eventual fate toward nonfunctionalization, subfunctionalization, or neofunctionalization. Thus, the immediate phenotypic and fitness consequences of duplications have not received the same degree of attention. Second, the fitness consequences of the *bar* mutation are most likely deleterious (Geer and Green, 1962). Although there is abundant evidence of beneficial duplications, particularly in the context of stressful or perturbed environmental conditions (Maroni et al., 1987; Theodore et al., 1991; Brown et al., 1998; Evgen’ev et al., 2004; Hemingway et al., 2004; Gonzalez et al., 2005; Deng et al., 2010; Nasvall et al., 2012; among others), changes in gene copy-number are usually deleterious (Lupski, 1998; Inoue and Lupski, 2002; Botstein and Risch, 2003; Bailey and Eichler, 2006; Sebat et al., 2007). Before the recent advances in detecting copy-number changes, an estimated 29% of human genetic diseases were thought to result from gene copy-number changes, with 22 and 7% stemming from gene deletions and duplications, respectively (Botstein and Risch, 2003). Lastly, Sturtevant and Morgan (1923) discovered that the segmental duplications that gave rise to the *bar* phenotype were unstable. Although the original experiments on the *bar *mutation do not provide an estimate of the rate of duplication, the frequency of reversions due to duplication loss and the frequency of double-*bar* mutation from *bar *flies was very high, on the order of approximately 10^-^^3^ per generation (Sturtevant, 1925). These early experiments with the* bar* mutation therefore showed that gene copy-number changes can occur at much higher rates than point mutations.

The study of structural genetic variation is undergoing an epochal resurgence. The reasons for this increased interest are largely technical. The explosive increase in the number of sequenced genomes has made it abundantly clear that the primary source of new genes is gene duplication, as previously advanced by Ohno (1970). Complementarily, high-throughput screens of structural variation in natural populations have demonstrated that there is abundant genetic variation in gene copy-number variation that we were previously unable to detect on a genome-wide scale (Iafrate et al., 2004; Sebat et al., 2004; Maydan et al., 2007; Emerson et al., 2008; among others). Finally, direct measurements of mutation rates have shown that structural genetic variation arises much more frequently than bioinformatic analysis of the age-distribution of extant duplicates in the first sequenced genomes had suggested (Lynch et al., 2008; Lipinski et al., 2011; Schrider et al., 2013). The high frequencies of spontaneous genome rearrangements and gene copy-number variants (CNVs) have important implications for the evolution of novel genes, speciation and hereditary disease. Much of the recent work in gene duplication has focused on gene copy-number polymorphisms in natural populations, and testing hypotheses of functional divergence between paralogs. Here, however, we review recent developments on two related topics regarding gene duplications, namely the spontaneous rate of segmental gene duplications and deletions, and their fitness consequences.

### THE FATE OF DUPLICATED GENES IN POPULATIONS

Although genomes can provide a rich record of the history of gene duplications in a particular lineage, the population-genetic dynamics and selection pressures on duplicated genes remain poorly understood. The frequency of gene copy-number polymorphisms in populations is determined by a combination of the spontaneous duplication/deletion rate and the preservation or elimination of these changes by natural selection and/or random genetic drift.

The fixation of a gene duplicate in a population faces multiple obstacles. First, there is a high probability that the duplicated gene is lost from the population by random genetic drift. Moreover, most gene duplications are probably detrimental to organismal fitness. They can perturb optimal dosage balance between genes contained in the duplicated regions with genes elsewhere in the genome, and increased gene dosage can be costly because of superfluous gene expression (Papp et al., 2003; Veitia, 2004). Empirical estimates of this cost in *Salmonella* was found to be substantial (3–16%; Reams et al., 2010). In addition to reducing fitness, many gene duplications are inherently unstable, particularly if they are in tandem orientation or flanked by repeat elements (Anderson and Roth, 1981). Lastly, given that most mutations are degenerative, a duplicated gene is much more likely to end up as a pseudogene than to acquire a function that is distinct from the ancestral gene and actively maintained by natural selection. Loss of one copy, either due to deletion or mutational inactivation is the fate of the overwhelming majority of duplicated genes (Haldane, 1933; Lynch and Conery, 2000). How redundant gene copies get to be fixed and subsequently maintained in a population has emerged as an important issue in the population-genetic theory of evolution by gene duplication (Force et al., 1999).

Several mechanisms have been proposed that would facilitate retention of a duplicated gene in a genome. (i) Redundancy could be beneficial because it protects the genome from the immediate deleterious effects of degenerative mutations (Clark, 1994). (ii) Degenerative mutations can lead to loss of different subfunctions in the two copies of a gene in such a way that both copies would be required to perform what was originally the role of a single ancestral locus (DDC, Duplication-Degeneration-Complementation; Hughes, 1994; Force et al., 1999). (iii) If there is a heterotic interaction (or overdominance) between alleles at a locus, the same beneficial interaction between alleles at two loci can maintain the duplication through natural selection (Spofford, 1969). (iv) Natural selection can result in functional divergence (neofunctionalization) between alleles prior to gene duplication and different alleles can then be preserved at different loci following duplication (Proulx and Phillips, 2006). (v) Although gene duplications create redundant gene copies, many detrimental mutations could still be subject to purifying selection if they interfere with the function of the wild-type copy and this would delay the process of turning one of the gene copies into a pseudogene (Walsh, 2003). However, selection against these detrimental mutations would not protect against the deletion of duplicated genes. (vi) Increase in gene dosage (“more of the same”) can be advantageous directly and would result in an increase in gene copy-number (Ohno, 1970). Selection for greater gene dosage does not have to be for the gene’s primary activity. When a promiscuous side-function of a gene becomes biologically valuable, selection for increase in gene dosage would help the spread and maintenance of a duplicated gene in the population until subsequent beneficial mutations result in a novel gene (Roth et al., 1996; Hendrickson et al., 2002; Hooper and Berg, 2003; Bergthorsson et al., 2007). There are certain similarities between some of these proposed mechanisms of selective retention of duplicates. For example, hypotheses (iii), (iv), and (vi), depend on natural selection for functions that are already present in the population prior to duplication.

### THE IMPORTANCE OF THE GENE DUPLICATION RATE IN EVOLUTION

The rate at which copy-number variation is introduced and eradicated from populations is crucial to understanding the early evolutionary dynamics of novel genes and the evolution of complexity. Both the standing levels of genetic variation and the genetic load are expected to be critically dependent on the rates and fitness effects of spontaneous gene duplications and deletions. The resolution of the duplication and deletion rate parameters will also serve to elucidate the role of gene copy-number in the evolution of disease.

The duplication rate is a key parameter in determining the equilibrium frequency of gene copy-number in populations. For neutral duplications, the equilibrium frequency of duplicated genes is expected to be *D*/(*D *+ *L*), with *D* as the spontaneous duplication rate and *L* as the rate of spontaneous loss of duplicate gene copies. In the event of deleterious duplications, the equilibrium frequency still depends largely on the duplication rate. The opportunity for mutations that result in the maintenance of duplicate copies, either through neofunctionalization or subfunctionalization, depend on the equilibrium frequency of additional gene copies in the population, and hence on the spontaneous gene duplication (and loss) rate. The duplication rate may therefore have profound effects on the role of adaptation in the evolution of duplicated genes (Ohta, 1988).

Following the rediscovery of Mendel’s laws, some geneticists started attributing greater importance to mutations as the driving force in evolutionary change, and de-emphasizing the importance of natural selection (Morgan, 1916, 1925). The importance of mutations and their rate as the greatest determining factor in evolution fell out of favor after it was shown that the mutation rate is, at best, a very weak force in effecting changes in allele frequency (Haldane, 1932, 1933). The neutral theory led to a greater appreciation of mutation rates as an evolutionary force, but primarily for neutral mutations (Kimura, 1983). More recently, theoretical and experimental evidence suggest that differences in mutation rates can have an orienting effect on evolutionary change (Yampolsky and Stoltzfus, 2001; Rokyta et al., 2005). Mutations are, in this view, not simply raw material for evolutionary change, but the differences in the rates of supply of different mutations influences the outcome with respect to adaptive evolutionary change. Given equal mutation rates, the mutations with the highest fitness contributions will, on average, be fixed first (Orr, 2003). However, mutations that are less fit can be fixed in the population earlier than the fittest mutation if the former are more frequent (Yampolsky and Stoltzfus, 2001; Rokyta et al., 2005). Moreover, the influence of the mutation rate on the rate of fixation of beneficial mutations is greater at smaller effective population sizes (Yampolsky and Stoltzfus, 2001). Let us consider the case of selection for increased gene dosage. Both gene duplication and point mutation can result in increased gene expression, and many point mutations might yield higher expression levels than duplications. However, if the gene duplication rate greatly exceeds the per nucleotide substitution rate, duplications will have an opportunity to increase in frequency, and perhaps reach fixation, before the appearance of point mutations in the population with similar or greater effects on gene expression. The rate of gene duplication relative to base substitutions is therefore particularly relevant for the hypothesis that selection for gene dosage is important in the initial preservation of duplicated genes.

### ANALYTICAL METHODS USED TO ESTIMATE THE GENE DUPLICATION AND DELETION RATE

Several approaches have been used to estimate the spontaneous gene duplication and deletion rates. These estimates have primarily come from four sources: (i) direct measurements on a single locus where gene copy-number differences resulted in a distinct phenotype or genotype, (ii) analyses of frequencies of duplication polymorphisms in populations, (iii) calculations based on the abundance of evolutionarily recent gene duplications in sequenced genomes, and (iv) direct genome-wide estimates of the duplication/deletion rate from molecular analyses of mutation accumulation (MA) lines evolved experimentally under a regime of minimal natural selection.

Direct estimates at specific loci have yielded the highest gene duplication frequencies. In contrast, analysis of the age distribution of genes in sequenced genomes yields rates that are orders of magnitude lower (Lynch and Conery, 2000, 2003; Gu et al., 2002; Pan and Zhang, 2007). However, the analyses of sequenced genomes assume that the birth and death rates of duplicated genes are constant over long evolutionary periods. This may be unwarranted if most gene duplications are detrimental and removed from the population by natural selection soon after conception.

## PER-LOCUS RATES

Per-locus rates of gene duplication have been empirically generated for bacteria, flies and humans (Table 1). However, these estimates are often based on a very limited number of loci and may not be representative for these genomes.

**Table 1:** Locus-specific duplication rates for prokaryotes and eukaryotes.

Species	Locus-specific duplication rates	
	Locus	Partial genome
**Prokaryotes**
*S. enterica *	2.0 × 10^-3^ (ArgH)^(a)^	3.2 × 10^-3^ – 5.8 × 10^-5^ duplications per locus^(b)^
	3.0 × 10^-4^ (LacZ)^(a)^	
	4.6 × 10^-6^(PyrD)^(a)^	
**Multicellular eukaryotes**
*D. melanogaster*	1.6 × 10^-5^(Rosy)^(c)^	
	1.7 × 10^-4^(Rosy)^(d)^	
	2.7 × 10^-6^(Maroon-like)^(d)^	
	4.0 × 10^-7^(Body- and eye-color)^(e)^	
*H. sapiens*	1.7 × 10^-5^(PMP22)^(f)^	
	2.6 × 10^-5^(α-globin)^(g)^	
	1.0 × 10^-8^(DMD)^(h)^	

*One rate estimate based on 38 loci is included. All rate measurements are in duplications/gene/generation unless otherwise specified. The loci are listed in parentheses.*

(a) Reams etal. (2010)

(b) Anderson and Roth (1981); across 38 loci in overnight culture

(c) Gelbart and Chovnick (1979)

(d) Shapira and Finnerty (1986)

(e) Watanabe etal. (2009)

(f) Lupski (2007)

(g) Lam and Jeffreys (2007)

(h) Van Ommen (2005)

### PROKARYOTES

Early experiments with phage and bacteria suggested a fairly high duplication rate per gene. For example, experiments with the *lac* operon in *Escherichia coli* suggested spontaneous duplications rates on the order of 10^-^^3^ to 10^-^^4^ per gene (Horiuchi et al., 1963; Langridge, 1969; Anderson and Roth, 1977). More generally, the reported frequency of duplication rates in bacteria and phage for a diversity of genes ranged from 10^-^^3^ to 10^-^^5^ (Anderson and Roth, 1977; Starlinger, 1977). The first systematic large-scale study of duplication frequency analyzed 38 duplicated loci in stationary phase cultures of *Salmonella* and found frequencies ranging from 10^-^^3^ to 10^-^^5^ per gene (Anderson and Roth, 1981). It should be noted that these estimates do not constitute duplication rates per generation as they had accumulated during the growth of the culture where the duplication rate had been countered by both a high rate of spontaneous duplication loss and natural selection. A more recent analysis of the duplication rate at three loci in the *Salmonella* genome found rates ranging from 2 × 10^-^^3^ to 4.6 × 10^-^^6^ duplications/gene/generation after carefully controlling for selection and spontaneous duplication loss (Reams et al., 2010). The equilibrium frequency of duplications in culture can likewise be quite high, and high-throughput sequencing of *Salmonella* cultures demonstrated that the percentage of cells carrying duplications had reached a steady-state frequency of 20% (Sun et al., 2012).

### EUKARYOTES

Direct estimates of duplication rates at two loci in *D. melanogaster*, the *maroon-like* and the *rosy,* were 2.7 × 10^-^^6^ and 1.7 × 10^-^^4^ duplications/locus/generation, respectively (Gelbart and Chovnick, 1979; Shapira and Finnerty, 1986). More recently, inverse PCR-based methods were used to measure the rates of duplication and deletion of human *α-globin* genes (Lam and Jeffreys, 2006, 2007). The frequencies of spontaneous *α-globin* duplication in sperm were 2.6 × 10^-^^5^ and 6.2 × 10^-^^5^ in two human males. However, it is possible that the actual duplication rate of *α-globin* genes is in fact higher than reported because the PCR primers used to detect the duplications were designed to detect specific kinds of duplications, and translocated and inverted duplications would not have been detected. Similar methods were used to determine the duplication and deletion rates at four loci in humans and the duplication rate estimates ranged from 1.7 × 10^-^^5^ to 8.7 × 10^-^^7^ (Turner et al., 2008).

Lastly, Watanabe et al. (2009) screened 1,554 progeny of wild-caught *D. melanogaster* females for spontaneous eye- and body-color mutations and identified five large deletions ranging from 40 to 500 kb. If these deletions originated via unequal crossing-over, the duplications rate should equal the deletion rate. Based on this assumption, the per gene duplication rate was estimated to be 4 × 10^-^^7^/generation, a similar order of magnitude as other empirical per gene duplication rates in *Drosophila* (Watanabe et al., 2009).

These estimates from single loci yield some of the highest estimates of the duplication rate. This may stem from both a sampling bias toward loci with known high duplication rates, and because some of the examples come from loci that are experiencing unequal crossing-over between related genes. For example, analysis of the duplication rate at the *rosy* locus was undertaken after observing that tandem duplications were occurring at an unusually high frequency (Gelbart and Chovnick, 1979). Similarly, *α-globin* gene copy-number polymorphism was well known and particularly common in populations with high exposure to malaria (Lam and Jeffreys, 2006). The high rate of duplications and deletions found in these systems may therefore not be representative of the genome at large.

## ESTIMATES OF THE DUPLICATION RATE BASED ON POPULATION FREQUENCY OF CNVs

The duplication rate can also be estimated using the frequency of gene duplications in a population and population-genetic theory of mutation-selection balance. Haldane (1935) showed that for X-linked genes in equilibrium, the mutation rate can be estimated using 1/3(1 - *f*)*x*, where *f* is the fertility of affected males relative to unaffected males and *x* is the frequency of affected males in the population. If the X-linked mutation results in lethality or sterility, the mutation rate is estimated as *x*/3. Using this approach, Van Ommen (2005) calculated the rate of new gene duplications in the X-linked human dystrophin gene leading to Duchenne Muscular Distrophy (DMD). Males with DMD have, until recently, been mostly nonreproductive. The frequency of DMD in male newborns is 1:3,500 and the frequency of mutations leading to DMD is thus ~10^-^^4^ (Table 1). Subgenic duplications account for 9% of these mutations and the rate of duplication was therefore estimated to be ~10^-^^5^ duplications/DMD locus/generation. The DMD is very large (2.5 Mb) and extrapolating from this region to the whole genome, the genome-wide duplication rate should be 0.02 duplications/genome/generation. This would be an underestimate if (i) many internal duplications do not result in a DMD phenotype, and/or (ii) if duplications that encompass the whole locus do not result in a DMD phenotype.

CMT1A, a subtype of Charcot-Marie-Tooth (CMT) syndrome, frequently results from a large duplication that includes the *PMP22* gene. Based on the prevalence of CMT1A and the fraction of CMT caused by duplications, the spontaneous duplication rate was estimated to be between 1.7 and 2.6 × 10^-^^5^ duplications/*PMP22 *locus/generation (Lupski, 2007). This rate is very similar to the rate estimated for DMD and three orders of magnitude higher than the spontaneous point mutation rate in humans.

## BIOINFORMATICALLY DERIVED ESTIMATES OF THE DUPLICATION RATE FROM WHOLE GENOME SEQUENCES

Lynch and Conery (2000, 2003) pioneered methods for estimating the duplication frequency in sequenced genomes from the age-distribution of duplicated genes based on the synonymous site divergence between gene paralogs. Their analyses found, for example, that duplications arise at a rate of 0.0011, 0.0028, 0.0025 per gene per 1% divergence at synonymous sites in the *D. melanogaster, Caenorhabditis elegans,* and *Saccharomyces cerevisiae* genomes, respectively (Lynch, 2007; Table 2). The spontaneous base substitution rate in these species has been measured as 55, 21, and 3.3 × 10^-^^10^ mutations/base pair/generation (Haag-Liautard et al., 2007; Lynch et al., 2008; Denver et al., 2009; Keightley et al., 2009; Schrider et al., 2013). If we utilize these rates to convert the historical gene duplication rate to frequency per gene per generation, the duplication rate would be 60.5, 58.8, and 8.25 × 10^-^^11^ in *D. melanogaster, C. elegans* and *S. cerevisiae,* respectively. These calculations assume that synonymous site changes are neutral, and in the event that there is some negative selection on synonymous sites, the per generation duplication rates would be overestimated. However, it was noted that the duplication rates inferred from the age distribution of gene duplicates might be underestimates for several reasons. (i) The assembly of whole genome sequences following shotgun sequencing may erroneously assume evolutionarily recent gene duplicates for redundant sequences of single-copy genes (Lynch and Conery, 2003). (ii) This particular analysis did not include paralogs in gene families possessing more than five members. The rates of spontaneous duplication and deletion might increase with the size of a gene family due to greater abundance of regions of high sequence identity that could serve as targets for copy-number changes by unequal exchange.

**Table 2:** Genome-wide estimates of the duplication rates for prokaryotes and eukaryotes.

Species	Genome-Wide Gene Duplication Rate	
	*Bioinformatic*	*Empirical*
**Unicellular eukaryotes**
*E. cuniculi*	11.7 × 10^-^^3^ per 1% silent-site divergence^(a, b)^	–
*P. falciparum*	0.3 × 10^-^^3^ per 1% silent-site divergence^(a, b)^	–
*S. cerevisiae*	2.5 × 10^-^^3^ per 1% silent-site divergence^(a, b)^	3.4 × 10^-^^6^^(d)^
	1.0 × 10^-^^11^/gene/year^(c)^	
*S. pombe*	1.6 × 10^-^^3^ per 1% silent-site divergence^(a, b)^	–
**Multicellular eukaryotes**
*A. gambiae*	6.2 × 10^-^^3^ per 1% silent-site divergence^(a, b)^	–
*A. thaliana*	3.2 × 10^-^^3^ per 1% silent-site divergence^(a, b)^	–
*C. elegans*	2.8 × 10^-^^3^ per 1% silent-site divergence^(a, b)^	3.4 × 10^-^^7^^(e)^
*D. melanog-* aster	1.1 × 10^-^^3^ per 1% silent-site divergence^(a, b)^	3.7 × 10^-^^7^^(f)^
*F. rubripes*	4.3 × 10^-^^3^ per 1% silent-site divergence^(a, b)^	–
*H. sapiens*	4.9 × 10^-^^3^ per 1% silent-site divergence^(a, b)^	–
	1.1 × 10^-^^9^/gene/year^(g)^	
*M. musculus*	3.0 × 10^-^^3^ per 1% silent-site divergence^(a, b)^	–

*Estimates are further classified into bioinoformatic versus empirical estimates. Bioinformatic estimates are based on the distribution of evolutionarily young gene duplicates in the genomes of laboratory strains or natural isolates. Empirical estimates are derived from mutation accumulation (MA) experiments involving experimental lines propagated under strict bottlenecking conditions. All rate measurements are in duplications/gene/generation unless otherwise specified. The loci are listed in parentheses.*

(a) Lynch and Conery (2003)

(b) Lynch (2007)

(c) Gao and Innan (2004)

(d) Lynch etal. (2008)

(e) Lipinski etal. (2011)

(f) Schrider etal. (2013)

(g) Cotton and Page (2005)

Gene conversion between duplicate gene copies lowers nucleotide sequence divergence between them, making them appear evolutionarily younger than they actually are (Teshima and Innan, 2004; Katju and Bergthorsson, 2010; Rane et al., 2010). If gene conversion between duplicated genes is common, the number of recent gene duplications in genomes is overestimated under the approach used by Lynch and Conery (2000, 2003). This in turn would lead to an inflated gene duplication rate. Using the genome of *S. cerevisiae* and six of it relatives, Gao and Innan (2004) calculated the gene duplication rate in yeast by a method that does not depend on synonymous site divergence between duplicate copies in a genome. They found strong evidence for gene conversion between duplicate gene copies, and estimated gene duplication rates to be 0.01–0.06 duplications/gene/billion years, two orders of magnitude lower than the previous estimate of Lynch and Conery (2000). However, *S. cerevisiae* with its large effective population size (*N*_e_ = ~3.3 × 10^7^; Lipinski et al., 2011) typically characteristic of unicellular eukaryotes is subject to a strong intensity of natural selection. Hence, the observed number of extant gene duplicates in a sequenced genome may grossly underestimate the gene duplication rate as many gene paralogs may have been purged from the genome in their infancy leaving no signature of their brief existence (Katju et al., 2009; Watanabe et al., 2009; Lipinski et al., 2011; Katju, 2012).

Codon usage bias due to selection for optimal codon use might also confound analyses of gene duplication rates with methods that rely on DNA sequence divergence at synonymous sites (Gu et al., 2002). The rate of molecular evolution in genes that are subject to natural selection against synonymous mutations in preferred codons is slower than at sites where nucleotide substitutions are selectively neutral. Duplicated genes that are experiencing selection for codon usage would therefore appear evolutionarily younger than they are. Gu et al. (2002) therefore suggested comparing DNA sequence divergence at synonymous sites in duplicated genes to sequence divergence in their introns and flanking sequences to exclude genes that appear to have undergone gene conversion or natural selection for codon usage bias. After “cleaning” their database of genes experiencing gene conversion or selection at synonymous sites, Gu et al. estimated the gene duplication rates in *S. cerevisiae*, *D. melanogaster,* and *C. elegans* to be 0.028, 0.0014, and 0.024 duplications/gene/million years, respectively. These results are qualitatively similar to the results of Lynch and Conery (2000, 2003).

More recently, Pan and Zhang (2007) estimated the gene duplication rates in mouse and humans, using synonymous site divergence as a proxy for the age of duplicated genes as some of the previous analyses, and attempting to distinguish between tandem duplications by unequal crossing over and retrotransposition. Their estimates of the overall gene duplication rate ranged from 0.0005 to 0.00149 and from 0.00123 to 0.00423 duplications/gene/million years in humans and mouse, respectively. Bensasson et al. (2003) arrived at similar rates as Lynch and Conery (2000, 2003) based on the number of duplicated mitochondrial genes that have been transferred to the nucleus (NUMTs).

## DIRECT GENOME-WIDE ESTIMATES OF THE SPONTANEOUS DUPLICATION RATE FROM MA EXPERIMENTS

Direct empirical analyses of individual loci where gene copy-number differences result in a distinct phenotype or genotype have provided the highest estimates of the gene duplication and deletion rates (Anderson and Roth, 1977, 1981; Shapira and Finnerty, 1986; Lam and Jeffreys, 2007; Watanabe et al., 2009). However, per-locus measures of the duplication/deletion rate may not be widely applicable at the genome-wide level. Experimental mutation accumulation (MA henceforth) lines in the estimation of mutation rates and parameters. First, they enable the most accurate estimation of mutation rates without the purging influence of purifying natural selection. Second, in conjunction with modern genome-wide techniques of analyses, they serve to directly quantify genome-wide mutation rates with minimal bias. The underlying principle behind MA experiments is straightforward; multiple replicate lines derived from an inbred ancestral stock population are allowed to evolve independently of one another under conditions of extreme bottlenecking each generation. The repeated bottlenecks severely diminish the efficacy of natural selection, promoting evolutionary divergence due to the accumulation of deleterious mutations by random genetic drift. The vast majority of MA studies have maintained the organism at a constant minimal *N*_e_ for the purpose of drastically reducing the efficacy of selection and enabling the accumulation of the vast majority of mutations (Mukai, 1964; Ohnishi, 1977; reviewed in Halligan and Keightley, 2009).

The advancement of molecular technologies such as high-throughput genome sequencing and oligonucleotide array comparative genome hybridization (oaCGH henceforth) have enabled genome-wide analyses of DNA content of MA lines to generate the first empirical measures of the spontaneous gene duplication and deletion rate in a handful of model organisms (Table 2). Lynch et al. (2008) conducted pulse-field gel electrophoresis (PFGE) and oaCGH on eight *S. cerevisiae* MA lines passaged through 200 bottleneck generations and estimated the spontaneous duplication rate to be 3.4 × 10^-^^6^ per gene/generation. This spontaneous duplication rate in *S. cerevisiae* is four orders of magnitude greater than the spontaneous base-substitution rate of 0.33 × 10^-^^9^ per site/generation in this species. Moreover, this spontaneous duplication rate vastly exceeds previous estimates arrived at from bioinformatic analyses (Lynch and Conery, 2000; Gao and Innan, 2004) of the originally sequenced *S. cerevisiae* genome (Goffeau et al., 1996). Additionally, the yeast genome originally sequenced by Goffeau et al. (1996) has an extremely low incidence of extant paralogs with low synonymous divergence that originated from small-scale duplication events (Katju et al., 2009). Of this already limited number of paralogs, a substantial number are likely of older evolutionary origin given the high incidence of selection for codon usage bias in conjunction with ectopic gene conversion within this species (Gao and Innan, 2004; Lin et al., 2006). So where are these new paralogs that are spawned at astoundingly high rates? One hypothesis is that most duplicates have, at the minimum, mildly deleterious fitness effects that renders them amenable to rapid purging from the genome in a unicellular eukaryotic species such as *S. cerevisiae* with a high *N*_e_ (Katju et al., 2009; Lipinski et al., 2011; Katju, 2012). As such, genome sequences of isolates/strains that have been subject to some degree of natural selection will invariably underestimate the spontaneous rate of duplication.

Lipinski et al. (2011) provided the first empirical, genome-wide estimates of the spontaneous rate of duplication and deletion in a multicellular eukaryote, the nematode *C. elegans*. As in the preceding study with *S. cerevisiae*, long-term MA lines formed the focus of this study to ensure unbiased estimates of the spontaneous rates of gene duplication with minimal influence of natural selection. Ten *C. elegans *MA lines subjected to single-worm bottlenecks for an average of 432 generations were assayed using oaCGH. In total, 14 duplicated segments that comprised *complete* and/or *partial *gene duplications were detected and verified independently via quantitative PCR. These duplicated segments encompassed 30 genes, giving a spontaneous rate of gene duplication of 3.4 × 10^-^^7^ per gene/generation for *partial* or *complete* duplications. If only *complete* gene duplicates were considered, the spontaneous rate of gene duplication was 1.25 × 10^-^^7^ per gene/generation. The authors argued that this estimate is downwardly biased for two reasons, namely (i) the number of adjacent microarray probes signaling gene copy-number changes may not be sufficiently dense for the detection of duplication events with small duplication spans, and (ii) the oaCGH DNA microarrays were restricted to unique probes only and duplications of genes in recently duplicated regions, for instance by unequal crossing over, may not have been detected. Despite the possibility that this rate is an underestimate, it is two orders of magnitude greater than the *C. elegans* spontaneous base-substitution rate of ~10^-^^9^ per site/generation (Denver et al., 2009). Additionally, this empirical spontaneous duplication rate estimate is two orders of magnitude greater than the estimate calculated from bioinformatic analyses of the frequency distribution of extant paralogs of varying evolutionary age (Lynch and Conery, 2000) in the originally sequenced genome of the *N2* laboratory strain of *C. elegans* (*C. elegans* Sequencing Consortium, 1998).

More recently, Schrider et al. (2013) sequenced the genomes of eight sublines derived from two ancestral lines of a long-term MA experiment in *D. melanogaster*. Despite the use of vastly different technologies for the estimation of the spontaneous duplication rate in *C. elegans* (oaCGH) and *D. melanogaster* (Illumina paired-ends sequencing), the duplication rate estimates are surprisingly similar. Schrider et al. (2013) generated the following rates for* D. melanogaster*: 3.75 × 10^-^^7^ per gene/generation for *partial* or *complete* duplications and 1.25 × 10^-^^7^ per gene/generation if only *complete *duplications were considered.

## ESTIMATES OF THE DELETION RATE

The frequency of gene copy-number polymorphisms in genomes is determined by a combination of the spontaneous duplication/deletion rate and the preservation or elimination of these changes by natural selection. Hence, in conjunction with other evolutionary forces such as selection and genetic drift, the net difference in the spontaneous rates of duplication and deletion has important consequences for the evolution of genome size. Furthermore, duplications and deletions may work in concert with one another. For example, aneuploidy and duplications were common in a collection of random yeast deletion mutants (Hughes et al., 2000). The duplicated regions often contained genes that were related to the deleted genes suggesting that the duplications were compensating for the deletions even though the primary functions of the deleted and duplicated genes are not identical (Hughes et al., 2000). There exists ample evidence that loss-of-function mutations, for example due to gene deletions, can often be suppressed or compensated for by multiple copies, or increased transcription of another gene in the genome (Berg et al., 1988; Bender and Pringle, 1989; Trempy and Gottesman, 1989; Ueguchi and Ito, 1992; Yamanaka et al., 1994; Serebrijski et al., 1995; Timms and Bridges, 1998; Menez et al., 2001; Miller and Raines, 2004; Patrick et al., 2007; Patrick and Matsumara, 2008). This phenomenon is known as “multicopy suppression” and typically results from side-functions of a multicopy gene that go unnoticed when it exists as a single copy in the genome (Berg et al., 1988). On the flip side, deletion events subsequent to duplications can occur commonly and pervasively at the genome-wide level, leading to the “diploidization” of polyploids and the evolution of reproductive incompatibilities (Wolfe, 2001; Kashkush et al., 2002; Langkjaer et al., 2003; Brunet et al., 2006; Scannell et al., 2006; Albertin and Marullo, 2012). Internal deletions of segmental duplications can also play a role in the eventual fate of duplications. Experiments with selected gene amplifications in S*almonella* have revealed that large duplications are frequently followed by internal deletions that appear to facilitate further amplification, by reducing the fitness cost associated with amplification of genes that are not under selection for increase in gene dosage (Kugelberg et al., 2006, 2010).

The gene deletion frequency in bacteria is generally lower than the duplication rate, and ranges from 10^-^^4^ to 10^-^^8^ (Starlinger, 1977). Using a combination of sequential bottlenecking of colonies which reduces effective population size and PFGE, experiments in *Salmonella* found the deletion rate to be 0.5 × 10^-^^8^ (Nilsson et al., 2005). This is probably an underestimate because there is still selection against deleterious deletions and the PFGE approach only detects relatively large deletions (Nilsson et al., 2005). If many deletions resulted in the loss of essential genes, they would not be represented in this estimate. However, if spontaneous gene deletion rates are indeed lower than gene duplication rates in bacteria, then what is keeping bacterial genomes lean? One contributing factor is adaptive gene loss (discussed below). We further need to take into consideration that the evolutionary dynamics of duplications are different from deletions in that duplications are prone to loss through recombination. Hence, the instability of segmental duplications relative to deletions likely serves as a factor in maintaining streamlined bacterial genomes. Lastly, natural selection in large bacterial populations is also expected to be more efficient in eliminating slightly deleterious duplications relative to multicellular eukaryotes with smaller effective population sizes.

Inverse-PCR methods in humans found that the duplication and deletion rates of *α-globin* were very similar. The frequency of deletions in *α-globin* genes can be common in areas where malaria is endemic, and polymorphism for the number of *α-globin* genes is probably maintained by balancing selection involving increased resistance to malaria (Flint et al., 1986). The frequencies of spontaneous *α-globin* deletions in the sperm of two human males were 1.6 × 10^-^^5^ and 6.8 × 10^-^^5^. More recently, similar methods were used to determine the duplication and deletion rates at four hotspots in human sperm and the deletion rate estimates ranged from 2.2 × 10^-^^5^ to 9.5 × 10^-^^6^, with all deletion rate estimates exceeding the duplication rates by 2.1 to 4.1 fold (Turner et al., 2008). The population frequency of CNVs resulting in DiGeorge-Velo cardiofacial syndrome, Williams-Beuren syndrome and Smith-Magenis syndrome have been used to estimate the spontaneous deletion rate in humans. The estimated rates range from 2 × 10^-^^5^ to 1.25 × 10^-^^4^ deletions/locus/generation (Lupski, 2007). Loss of gene duplication occurs generally at a higher rate than the duplication rate. For example, loss of the *bar *duplication in *D. melanogaster* may occur at a rate as high as 10^-^^3^ (Sturtevant, 1925).

Genome-wide estimates of the spontaneous deletion rates are currently available for three species: *S. cerevisiae* (Lynch et al., 2008), *C. elegans* (Lipinski et al., 2011) and *D. melanogaster *(Schrider et al., 2013). The spontaneous deletion rates were 2.1 × 10^-^^6^, 2.2 × 10^-^^7^, and 9.37 × 10^-^^7^/gene/generation in *S. cerevisiae*, *C. elegans,* and *D. melanogaster*, respectively. In *S. cerevisiae* and *C. elegans*, there appears to be a slight excess of duplications relative to deletions when considered on a gene-by-gene basis, whereas the deletion rate exceeded the duplication rate in the *D. melanogaster* experiment. However, deletions tend to be smaller than duplications and the net change in base pairs is positive in all three experiments. That is, nucleotides added by duplications exceed those deleted.

## FITNESS EFFECTS OF CNVs

The scientific literature is replete with descriptions of gene duplications that are either beneficial or detrimental to the fitness of their carriers. On the beneficial side, some of the most striking examples in humans include the copy-number increase of the human salivary amylase gene (*AMY1*) that have enabled adaptation to a high-starch diet (Perry et al., 2007) and copy-number increase of the *CCL3L1* gene that is associated with lowered susceptibility to HIV infection (Gonzalez et al., 2005). Interestingly, the domestication of dogs by humans too has resulted in a copy-number increase in the canid *amylase* gene, enabling dogs to benefit from a high-starch diet that is distinctly human and contrasting from their wolf ancestors (Axelsson et al., 2013). Copy-number increases are also implicated in adaptation to novel or resource-limited environments in microbial laboratory populations (Sonti and Roth, 1989; Reams and Neidle, 2003), insecticide resistance (Newcomb et al., 2005) or metal tolerance (Maroni et al., 1987) in natural insect populations, drug resistance in parasites (Nair et al., 2007), increased vertebrate resistance to bacterial pathogens (Jackson et al., 2007) and as a compensatory response to loss-of-function mutations (Berg et al., 1988; Bender and Pringle, 1989; Trempy and Gottesman, 1989; Ueguchi and Ito, 1992; Yamanaka et al., 1994; Serebrijski et al., 1995; Timms and Bridges, 1998; Menez et al., 2001; Miller and Raines, 2004; Patrick et al., 2007).

However, most gene duplications are probably deleterious. The detrimental consequences of duplications can come from a variety of sources: (i) dosage imbalance between the duplicated genes and other genes in the genome that remain in single copy, (ii) inappropriate expression of gene duplicates that are under the control of a different regulatory system, and (iii) the cost of superfluous expression. From the perspective of the deleterious nature of gene duplications, increases in gene copy-number are implicated in increased susceptibility to a wide range of human diseases (Lupski, 1991, 1998; Inoue and Lupski, 2002 and references therein; Botstein and Risch, 2003; Sebat et al., 2007). Several additional lines of evidence support the notion that gene duplications are, on average, deleterious. First, the large discrepancy in empirical (from MA experiments) and bioinformatics-based estimates of the gene duplication rate is best explained by selection against new duplications (Katju et al., 2009; Lipinski et al., 2011). Bioinformatically based methods to determine the duplication rate from the age distribution of genes in a sequenced genome assume a constant loss rate for duplicate genes. However, if selection against duplicate copies in their infancy removes most detrimental gene duplicates before they can diverge at the DNA sequence level, the loss rate may appear to be constant, and yet result in an underestimate of the spontaneous duplication rate. Second, population variation in gene copy-number also suggests that duplications are generally detrimental. In natural populations of *D. melanogaster*, the allele frequencies of duplications are lower than expected if the duplications are neutral (Langley et al., 2012), although not all studies can reject the null hypothesis of no fitness consequences of completely duplicated genes (Emerson et al., 2008). Third, there is a negative correlation between allele frequencies of duplicates and recombination rates, which is consistent with the notion that greater efficacy of natural selection associated with higher recombination rates is eradicating duplicates at a greater rate from regions of high recombination relative to regions of low recombination (Langley et al., 2012). A significant negative association between the length of the duplicated segment and gene density with allele frequencies in humans and *Drosophila* (Itsara et al., 2010; Langley et al., 2012) suggests that duplications encompassing more genes are more deleterious than those spanning fewer genes. This is expected if dosage imbalance plays a large role in determining the fitness cost of duplications.

Deletions, like duplications, can be either detrimental or adaptive. Examples of adaptive deletions are more limited relative to adaptive duplications and it is generally assumed that deletions are, on average, more detrimental than duplications. Several genome-wide studies of copy-number variation in humans have found deletion alleles to occur in lower frequencies than duplication alleles (Conrad et al., 2006; Locke et al., 2006). This is suggestive of strong purifying selection weeding out deletions. Furthermore, a deficit of genic deletions has been observed in humans (Conrad et al., 2006, 2010; Redon et al., 2006) and *D. melanogaster* (Emerson et al., 2008; Langley et al., 2012), implying that deletions in coding sequences are more deleterious than duplications of these sequences, and therefore more likely to be purged by purifying selection. Conrad et al. (2010) compared the relative frequencies of deletions in two additional genomic regions, namely intronic and intergenic. Intergenic deletions outnumbered intronic deletions, suggesting stronger selection against the latter, given their central role in the maintenance of accurate intronic sequence for splicing (Conrad et al., 2010). This might also explain why the frequency of spontaneous deletions appears lower than duplications in MA experiments in yeast and *C. elegans* (Lynch et al., 2008; Lipinski et al., 2011). Although MA experiments can capture a wide range of deleterious mutations, mutations with severe fitness consequences are still less likely to be fixed than mutations with minor and moderate fitness costs.

Nonetheless, deletions have played an important role in adaptation. For example, a recurrent deletion of an enhancer for *Pitx1* in sticklebacks is associated with adaptive pelvic reduction (Chan et al., 2010). Adaptive deletions might be more common than we assume. In experiments with *Salmonella*, a surprisingly high proportion of deletions resulted in increased growth rate, which suggests that many bacterial genes are not necessary, and indeed a burden, in a specific laboratory environment (Koskiniemi et al., 2012). Parallel patterns of gene loss have been seen in bacteria, for example, during infection or host adaptation and although it is tempting to ascribe these to adaptive gene loss, these patterns can, in principle, also be explained by relaxation of selection on the lost genes (Feng et al., 2011; Rau et al., 2012). However, many studies of bacterial genome evolution suggest that gene loss is often adaptive. For example, the removal of pseudogenes from *Salmonella* genomes occurs at a faster rate than expected if the gene loss is purely neutral (Kuo and Ochman, 2010). The question of whether deletions are beneficial or neutral is easiest to address in an experimental setting rather than by retrospective analysis. In experiments with *Methylobacterium*, Lee and Marx (2012) found that repeated gene loss was adaptive, and the benefit from the deletions was not due to a shorter genome *per se*. The frequent and parallel patterns of gene loss in bacterial genomes recently inspired the Black Queen Hypothesis, which suggests that the evolution of dependencies in microbes resulted from selection against genes whose products can be acquired from other organisms (Morris et al., 2012).

## THE ROLE OF *N*_e_ IN DICTATING CNV LOSS OR FIXATION

The loss or fixation of CNVs and their consequences for population fitness depend upon both (i) the selection coefficients (*s*) associated with individual duplications/deletions, and (ii) the effective population size (*N*_e_) for the species. The fate of duplications/deletions with selection coefficients much less than the reciprocal of the *N*_e_ [∣ *s*∣ ≪ 1/2*N*_e_ for diploids] are expected to be dictated entirely by random genetic drift. Conversely, the dynamics of duplications/deletions with ∣ *s*∣ ≫ 1/2*N*_e_ are governed by natural selection. Deleterious duplications and deletions with very large deleterious effects will be rapidly eradicated from the population and unlikely to reach fixation; those with very small effects would be effectively neutral. Although the effect of any mutation is dependent on the *N*_e_, the prevailing opinion is that the most detrimental class of mutations influencing long-term population fitness includes mutations with small selection coefficients, also referred to as slightly deleterious or nearly neutral mutations (Ohta, 1992). Such mutations would be eradicated via purifying selection at high* N*_e_, but can behave in an “effectively neutral” fashion and reach fixation by genetic drift at low *N*_e_ (Lynch and Gabriel, 1990; Lande, 1994).

Empirical estimates of the spontaneous duplication rate, be they locus-specific or genome-wide from MA studies, invariably exceed estimates from analyzing the age distribution of gene duplicates in sequenced genomes. What may explain this discrepancy, with empirical estimates exceeding bioinformatically based ones by two to four orders of magnitude? We have previously proposed that the degree of discrepancy in bioinformatic and empirical estimates of the gene duplication rate is influenced by differences in the efficacy of selection in species due to their varying* N*_e_ (Katju et al., 2009; Lipinski et al., 2011; Katju, 2012). Specifically, slightly deleterious CNVs will be efficiently weeded out in species with large *N*_e_ but are more likely to survive the onslaught of purifying selection in species with small *N*_e_. Currently, bioinformatic and spontaneous empirical estimates of the gene duplication rate are only available for three species, *S. cerevisiae*, *D. melanogaster* and *C. elegans* with estimated *N*_e_ of 3.3 × 10^7^, 1.15 × 10^6 ^and 80,000 individuals, respectively (Lipinski et al., 2011; Katju, 2012 and references therein). The empirical estimates of the duplication rate exceed the bioinformatic estimates by 36,000-, 660-, and 340-fold for *S. cerevisiae*, *D. melanogaster,* and *C. elegans*, respectively. This discrepancy correlates positively with the species *N*_e_ as we have previously predicted (Lipinski et al., 2011). A more robust test of this hypothesis will require greater sampling of the empirical genome-wide duplication rates across more species.

## CONCLUDING REMARKS

Gene CNVs are of fundamental importance for genetic variation in populations, genome evolution and the evolution of genes with novel functions. When the first genome-wide estimates of the spontaneous duplication rate were bioinformatically determined from sequenced genomes, they were reported as being similar to the point mutation rates (Lynch and Conery, 2000). These rates were hailed as being “astronomical” (Pennisi, 2000). Direct empirical estimates of spontaneous duplication rates derived from experimental MA lines have been demonstrated to be orders of magnitude higher. The discrepancy between the bioinformatically derived and empirical duplication rates suggests that the vast majority of gene duplications are deleterious and rapidly eradicated from genomes before being afforded any opportunity to impart a genomic signature of their all too brief existence. This discrepancy between bioinformatically and empirically derived estimates of the duplication rate also appears to be positively correlated with the species *N*_e_. Prokaryotes and unicellular eukaryotes with large *N*_e_ and greater efficacy of selection are expected to rapidly purge even mildly deleterious duplicates. Conversely, in organisms with small *N*_e_ such as many multicellular eukaryotic species, genetic drift is expected to play an integral role in the accumulation of gene duplicates leading to the eventual preservation of duplicates following functional divergence.

The last decade or so has witnessed a revolution in the cataloging of structural variants in species, both at the population- and genomic-level. Structural variants, however, present multiple challenges in the analysis of their dynamics in populations and the evolutionary forces responsible for their ultimate fate in genomes. Whereas standard population-genetic theory is well-equipped to analyze the frequency of alleles or base substitutions in populations, CNVs of particular genes can have breakpoints in different locations, and duplicated genes can have additional variation with respect to genomic location and transcriptional orientation, all of which can differentially influence their function. In this review, we have not tackled issues relating to the structural complexity of CNVs. Gene duplicates, for example, exhibit varying degrees of structural resemblance to their progenitor loci (Katju and Lynch, 2003, 2006; Katju, 2012). An advanced understanding of how the structural resemblance between paralogs influences their eventual fate (pseudogenization, subfunctionalization, or neofunctionalization) must precede and is germane to elucidating the full contribution of CNVs to genome evolution.

Although most CNVs appear to be selected against, we need more information about their distribution of fitness effects, and what particular aspects of their genomic and molecular structure underlie these phenotypic fitness costs/gains. Are duplication and deletion rates species-specific and if so, do these show a dependence on the structural features of a genome, say the fraction of repetitive sequences within a genome? Furthermore, how do these high rates of duplication influence the fate of duplicated genes in populations via natural selection or genetic drift. One consequence of a high duplication rate is that adaptive variation in gene dosage can frequently arise by duplications. One of the important questions regarding the evolution of novel genes is how often this kind of selection for higher gene dosage results in functional divergence, for example, because of adaptive enhancement of subfunctions or promiscuous activity. Or is selection for gene dosage just a temporary response to ephemeral environmental challenges and do duplicates revert back to existence in single-copy form when these challenges no longer exist?

## Conflict of Interest Statement

The authors declare that the research was conducted in the absence of any commercial or financial relationships that could be construed as a potential conflict of interest.

## AUTHOR CONTRIBUTIONS

Vaishali Katju and Ulfar Bergthorsson contributed equally to all aspects of designing and writing this manuscript.
